# THAP1 Mutations and Dystonia Phenotypes: Genotype Phenotype Correlations

**DOI:** 10.1002/mds.25146

**Published:** 2012-08-17

**Authors:** Georgia Xiromerisiou, Henry Houlden, Nikolaos Scarmeas, Maria Stamelou, Eleanna Kara, John Hardy, Andrew J Lees, Prasad Korlipara, Patricia Limousin, Reema Paudel, Georgios M Hadjigeorgiou, Kailash P Bhatia

**Affiliations:** 1 Department of Molecular Neuroscience and Reta Lila Weston Institute, University College London Institute of NeurologyLondon, London, United Kingdom; 2 Sobell Department of Motor Neuroscience and Movement Disorders, University College London Institute of NeurologyLondon, United Kingdom; 3 Unit of Functional Neurosurgery, Sobell Department of Motor Neuroscience and Movement Disorders, University College London Institute of Neurology, University College LondonLondon, United Kingdom; 4 Department of Neurology, Faculty of Medicine University of ThessalyLarissa, Greece; 5 Taub Institute, Sergievsky Center, Department of Neurology, Columbia UniversityNew York, New York, USA; 6 Department of Neurology, Medical School of National and Kapodistrian University of AthensAthens, Greece

**Keywords:** THAP1, dystonia, DYT6, mutations, phenotype, genotype

## Abstract

*THAP1* mutations have been shown to be the cause of DYT6. A number of different mutation types and locations in the *THAP1* gene have been associated with a range of severity and dystonia phenotypes, but, as yet, it has been difficult to identify clear genotype phenotype patterns. Here, we screened the *THAP1* gene in a further series of dystonia cases and evaluated the mutation pathogenicity in this series as well as previously reported mutations to investigate possible phenotype-genotype correlations. THAP1 mutations have been identified throughout the coding region of the gene, with the greatest concentration of variants localized to the *THAP1* domain. In the additional cases analyzed here, a further two mutations were found. No obvious, indisputable genotype-phenotype correlation emerged from these data. However, we managed to find a correlation between the pathogenicity of mutations, distribution, and age of onset of dystonia. THAP1 mutations are an important cause of dystonia, but, as yet, no clear genotype-phenotype correlations have been identified. Greater mutation numbers in different populations will be important and mutation-specific functional studies will be essential to identify the pathogenicity of the various THAP1 mutations. © 2012 *Movement* Disorder Society

Primary dystonias are often inherited as monogenic traits, but the inheritance can be complicated by reduced expression and phenocopies within families. Originally, dystonia was classified purely on clinical features, but the classification and understanding of the dystonias has grown dramatically over the last decade with the application of genetic testing. At least 17 distinct, likely Mendelian primary dystonias have been identified.^1^–^11^

The most common inherited dystonia is DYT1, encoded by the *TorsinA*^8^ gene. The DYT1 phenotype has been described extensively with variable clinical presentation and progression. A three-nucleotide deletion (GAG) in exon 5 accounts for almost all DYT1 cases. Three other mutations have been described in TorsinA in isolated cases and, with their pathogenicity, are still unclear for two of them.^12^–^14^ *THAP1*-associated DYT6 patients present with a wide variety of sites of onset and severity. Missense, nonsense, and frameshift mutations have been described in all three exons of the *THAP1* gene, but they tend to be concentrated in the THAP domain. THAP1 binds to the core promoter of TorsinA, and wild-type THAP1 represses the expression of TorsinA, whereas Dyt6-associated mutant THAP1 results in decreased repression of TorsinA and increased expression.^15^–^21^ Recent studies have emphasized the fact that THAP1 mutations occur frequently with oromandibular and laryngeal dystonia, but focal, segmental, and generalized dystonia have all been described. There have been no reported THAP1 genotype/phenotype predictors that have been associated with the range of clinical features.

Therefore, we screened the *THAP1* gene in a further series of dystonia cases from the United Kingdom and performed a meta-analysis of published cases to investigate a possible genotype/phenotype in THAP1-associated dystonia.

## Patients and Methods

### Patients and Screening of the THAP1 Gene

The *THAP1* gene was analyzed in a group of 150 DYT1-negative, characterized patients with various forms of primary dystonia. All patients gave informed consent, and ethics approval was obtained from the joint medical and ethics committee at the National Hospital for Neurology and Neurosurgery (07/Q0502/2). Patients were assessed and followed up by movement disorder specialists. The *THAP1* gene was analyzed in this series by Sanger sequencing, as previously described (RefSeq NM_018105.2).^19^ The control series that were sequenced consisted of 176 healthy UK white individuals who were older than 50 years and neurologically healthy, 40 North London Jewish controls, and 68 Indian control individuals.

### Review of Published THAP1 Data

A comprehensive literature review of all reported THAP1 mutations was performed to include as many patients as possible. Data were analyzed from 100 patients published in the literature since the discovery of DYT6-associated dystonia until August 2011.^22^–^35^

### Evaluation of Pathogenicity of Mutations Using Computational Prediction

Two algorithms were used to evaluate the effect of amino-acid substitutions on the function of THAP1: polymorphism phenotyping (polyphen) and sorting intolerance from tolerance (SIFT).^36^ Prediction is based on empirical rules applied to the sequence, phylogenetic, and structural information characterizing the amino-acid substitution (http://genetics.bwh.harvard.edu/pph/ and http://sift.jcvi.org). The Human Splicing Finder tool^37^ was used to evaluate mutations that potentially affect splicing (http://www.umd.be/HSF/).

### Statistical Analysis

We used descriptive statistics to present demographic, clinical, and other characteristics of patients overall and by dystonia type. Demographic and clinical characteristics of patients by dystonia type were examined using analysis of variance (Scheffe's post hoc) for continuous variables and the chi-square test for categorical variables. We calculated Cox's proportional hazards models with dystonia as the outcome and dystonia age of onset as the time-to-event variable. In an initial model, dystonia type was the time-constant predictor (generalized dystonia as the reference). In a subsequent model, we considered pathogenicity of the mutation (benign as the reference) as the time-constant predictor. IBM/SPSS software was used (*version 19*; SPSS, Inc., Chicago, IL).

## Results

Clinical details of patients are presented in Table 1. Among patients with dystonia that we screened, we identified three variants. These included one novel nonsense mutation p.R29X (c.85C>T) and one missense mutation:p. N136S:(c.407A>G) in the heterozygous and in homozygous state, respectively. The homozygous case was found to have been included in a previous study.^19^ None of these variations were found among control chromosomes.

**TABLE 1:** Clinical detais of patients with dystonia screened for THAP1 mutations

Patients	n = 150
Women (%)	80 (53)
Median age at onset, years (range)	28 (2–54)
Median age at examination, years (range)	39 (14–58)
Median duration of dystonia, years (range)	25 (0–40)
Distribution of dystonia (%)	
Focal	35 (23)
Segmental	53 (35)
Multifocal	22 (15)
Generalized	40 (27)

### Patient 1

The patient that carries the R29X mutation is a 27-year-old woman with generalized dystonia, which affects her arms, trunk (mildly), and both feet. She also has marked oromandibular dystonia with protrusion of the tongue. Her symptoms began around the age of 7. Initially, her leg was affected, then her handwriting, and, at the age of 11, her speech was involved. Although her tongue was markedly affected, swallowing was preserved. There was no family history.

### Patient 2

The patient that carries the N136S mutation in the heterozygous state is a 36-year-old right-handed woman that noticed a pain in her neck at the age of 30, which got worse over the next 4 years. At the age of 34, she noticed a bit of a head shake and she developed a right torticollis. There is no evident dystonia in other parts of her body. There is no family history of dystonia. She responds well to botulinum toxin injections.

### Literature Review

**FIG. 1:**
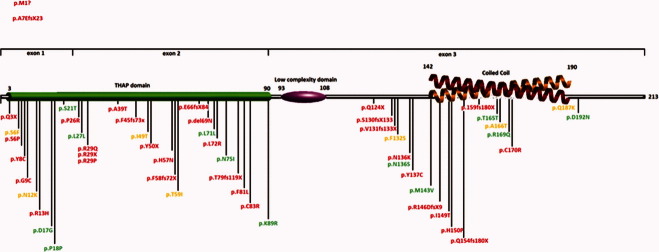
THAP1 mutations distributed throughout the gene. Each color represents different pathogenicity according to polyphen prediction program. Red: probable pathogenic; Yellow: possibly pathogenic; green: benign. [Color figure can be viewed in the online issue, which is available at wileyonlinelibrary.com.]

One hundred patients (60 females and 40 males) were included in our study group. In these patients, 63 different mutations have been identified (Fig. 1). The majority of these mutations are missense (66%), and the rest of them are small insertions/deletions, nonsense mutations, and splice-site mutations. In total, 77% of these were found to be probably and computationally possibly pathogenic, and 23% of them were computationally benign. The THAP domain (exons 1 and 2) contained 66% of mutations (Fig. 1). All descriptive characteristics of our study group are given in Table 2. We further analyzed these patients according to type of dystonia (Table 2).

**TABLE 2:** Clinical characteristics of patients published in the literature with THAP1 mutations and classification of mutations according to computational prediction

					First Symptom (%)				Pathogenicity of Mutation (%)		
						
	Mean Age at Onset Years (SD)	Family History (%)	Gender Distribution (Females) (%)	Speech Involvement (%)	Limb	Cervical	Cranial	Laryngeal	Benign	Possibly Damaging	Probably Damaging
Patients (total number = 100)	24.4 (18.6)	62	60	58	44	31	15	10	23	11	66
Type of dystonia											
Generalized (37%)	11.7 (8.7)	78.4	56.8	54.1	62.2	18.9	13.5	5.4	2.7	13.5	83.8
Segmental (30%)	24.7 (16.5)	56.7	50	50	40	30	20	10	23.3	6.7	70
Multifocal (6%)	19 (19)	100	66.7	33.3	100	0	0	0	0	16.7	83.3
Focal (27%)	42.8 (15.7)	37	77.8	18.5	11.1	55.6	14.8	18.5	55.6	11.1	33.3

SD, standard deviation.

**FIG. 2:**
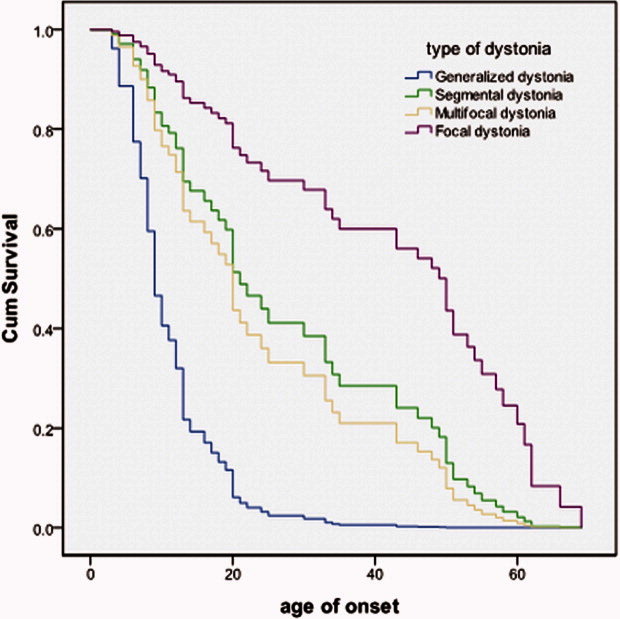
Survival curves based on Cox's analysis comparing DYT6 dystonia age of onset in patients grouped by dystonia type. Y-axis: 1-cumulative survival/probability of developing DYT6 dystonia. [Color figure can be viewed in the online issue, which is available at wileyonlinelibrary.com.]

An important result of our study is the effect of age at onset on the distribution of dystonia. Compared to patients with generalized dystonia, those with segmental (hazards ratio [HR] = 12.93; 95% confidence interval [CI] = 3.13–22.7; *P* = 0.004), multifocal (borderline, possibly resulting from a very small number of cases; HR = 7.27; 95% CI = −5.03−24.0; *P* = 0.07), and focal (HR = 31.08; 95% CI = 20.98–41.19; *P* = 0.000) dystonia had later age of onset (Fig. 2).

**FIG. 3:**
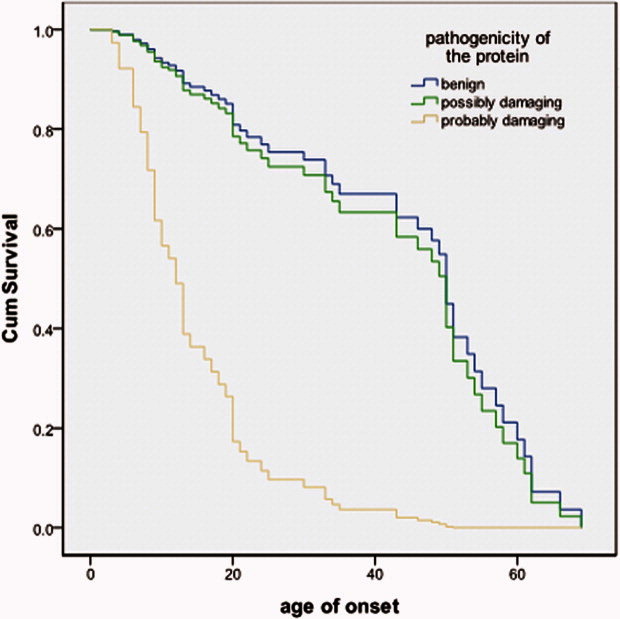
Survival curves based on Cox's analysis comparing DYT6 dystonia age of onset in patients grouped by mutation pathogenicity. Y-axis: 1-cumulative survival/probability of developing DYT6 dystonia. [Color figure can be viewed in the online issue, which is available at wileyonlinelibrary.com.]

Overall, no significant differences were observed between groups of patients with different types of dystonia and gender. In focal dystonia, though, we noticed that 77.8% of patients are women and 22.2% are men and that more benign variants have been found in women (70%), compared to men. In the generalized type of dystonia, 83.8% of patients harbor likely damaging mutations versus 4.3% that present with benign variants. On the other hand, in focal dystonia, 65.2% harbor benign variants versus 13.6% with likely damaging mutations (*P* < 0.0001). Limb onset is also associated significantly with more-pathogenic mutations (77.7% likely damaging versus 9.1% benign) versus cervical onset that benign and likely damaging mutations share the same percentage (41.9% and 48.4%, respectively). Compared to benign mutations, those with possibly damaging mutations had a slightly earlier age of dystonia onset (HR = 13.9; 95% CI = 1.07-26.8; *P* = 0.03), whereas those with probably damaging mutations had much earlier age of onset (HR = 28.8; 95% CI = 20.3-37.4; *P* = 0.000) (Fig. 3).

## Discussion and Conclusions

We have sequenced the *THAP1* gene in a series of British dystonia patients as well as analyzing the previous THAP1 reports. Two mutations were identified, consistent with previous reports and at a similar frequency of 1% to 2% of dystonia cases. This work shows that there were no specific *THAP1* mutations that consistently led to a severe or mild dystonia phenotype. This is the result of the large variety of THAP1 mutations that have been described, with few in more than one families and only one, the F45fs73X mutation, in a significant number of cases.^23^, ^28^

However, it is clear that THAP1 mutations influence several characteristics that include distribution of dystonia and age at onset. Patients with computationally pathogenic mutations, based on SIFT and POLYPHEN, frequently have generalized dystonia with earlier age at onset and positive family history. In a more simplistic way, this means that the probability of developing dystonia, that expresses survival endpoint, at a younger age is higher for patients with computationally pathogenic mutations, compared to the other groups. However, *in silico* investigations are not as good as true functional investigation.^38^ Prediction is based on factors such as three-dimensional protein structure, polarity, homology, and species conservation. These programs do not take into account other important factors, such as supramolecular interactions with homologous molecules.^38^ Overall, a correct phenotype is predicted in almost 70% of the cases, showing that the level of confidence is relatively high for research purposes.^39^

In a large number of cases with *THAP1* mutations, cervical dystonia was the presenting feature, in complete accord with all previous studies.^36–38^ More specifically, we observed that cervical dystonia usually emerges in middle age, is sporadic, and develops primarily in females. However, a large number of cervical dystonia cases harbor computationally benign variants. The main question remains: Can these variants cause the disorder or are they rare benign variants that have nothing to do with the dystonia. In most adult-onset dystonia families, inheritance does not appear to be Mendelian, but is rather consistent with a multifactorial trait.^41^ The main hypothesis today is that a number of common genes underlie the pathophysiological mechanisms shared by the various forms of adult-onset focal dystonia, and that additional genes and environmental triggers determine the clinical, neurophysiological, and imaging differences described in the various forms of dystonia.

To tackle the difficulty with the small number of cases, ideally, each mutation would be functionally characterized to investigate the degree of transcriptional dysregulation of TorsinA and the other consequences of a defective THAP1 protein.^16^ The analysis of further mutations and families will also be important, and recently, a web-based database has been created to allow the inclusion of mutation reports to increase the THAP1 dataset.^43^ More clinical and molecular data will be needed to elucidate the complex genotype/phenotype correlations associated with DYT6.
